# The role of family background on adolescent khat chewing behavior in Jazan Region

**DOI:** 10.1186/1744-859X-12-16

**Published:** 2013-05-20

**Authors:** Mohamed Salih Mahfouz, Rashad Mohammed Alsanosy, Abdelrahim Mutwakel Gaffar

**Affiliations:** 1 Family and Community Medicine Department, Faculty of Medicine, Jazan University, PO Box 2531, Jazan 45142, Saudi Arabia; 2 Substance Abuse Research Center (SARC), Jazan University, Jazan 45142, Saudi Arabia

**Keywords:** Khat chewing, Family background, Family attitude

## Abstract

**Background:**

Khat is a well-known natural stimulant from the *Catha edulis* plant and is widely used in certain Red Sea countries, including Yemen and the province of Jazan in Saudi Arabia. Jazan is located in the southwestern part of the Kingdom of Saudi Arabia adjacent to Yemen, where the practice of khat chewing is deeply rooted throughout the entire population. The main objective of this paper was to assess the association between family background, i.e., parent and sibling khat use, and adolescents' khat chewing behavior in Jazan. Other variables were also tested for association, including parents' education levels, family income, and peer influence.

**Material and methods:**

A cross-sectional study was conducted with a representative sample (*n* = 4,100) of intermediate and upper secondary school students of Jazan. The participants were selected using a three-stage cluster random sampling. A structured self-administered questionnaire was used for data collection. Descriptive statistics, a chi-squared test, and logistic regression were performed to examine the associations and predictors of khat chewing.

**Results:**

A total of 3,923 students of both genders from 72 intermediate and upper secondary schools in Jazan were involved in this study. Of these participants, 42.8% (1,678) were from intermediate schools and 43.8% (1,717) were females. The prevalence of current khat chewing among the students was 20.5% (95% confidence interval (CI) 19.27–21.79) and was significantly higher for males at 33.1% (95% CI 31.16–35.08) than for females, of whom 4.3% (95% CI 3.39–5.31) (*P* < 0.001) chew khat. The multivariate logistic regression analysis suggests that the most important independent predictors of student khat chewing included the students' smoking status (odds ratio (OR) = 14.03, *P* < 0.001), a friend using khat (OR = 5.65, *P* < 0.001), a sister using khat (OR = 2.04, *P* < 0.05), a father using khat (OR = 1.45, *P* < 0.001), and a brother using khat (OR = 1.56, *P* < 0.05).

**Conclusion:**

The results highlight the significant impact of peer and familial khat abuse in adolescent khat chewing behavior. The findings suggest that khat control programs need to focus on peers and family members to reduce the prevalence of the habit along with its unfavorable consequences.

## Background

The consumption of khat is widespread in East Africa and the southern portion of the Arabian Peninsula [1-3]. It is well documented that frequent khat chewing has been associated with unfavorable socioeconomic, public health, and environmental outcomes [4-6].

High-risk adolescent behaviors associated with substance abuse result from multiple causes, often beginning in early childhood, that change with age and are interrelated in complex ways. These causes operate at the levels of socioeconomic status, neighborhood, cultural context, peer influence, teachers' influence and, perhaps most importantly, family influences [7-9].

Three broad categories of family influence have been studied in the literature on adolescent risk-taking: the shape and quality of family interactions, parenting styles and practices, and family modeling and socialization of risky behaviors. Additional family characteristics such as parents' socioeconomic status, maternal age at the birth of the child, ethnicity, and family size and structure play contributing roles as well [7-11].

The literature also suggests that family factors and backgrounds play an important role in the development of youth behavior; this family role is very clear in smoking and other substance abuse [12-15]. However, few studies have investigated the role of family factors such as family members' khat chewing habits on adolescent behavior.

Traditional khat use is common in all segments of the population of Jazan province in the southwest area of the Kingdom of Saudi Arabia (KSA) [16,17]. Although khat chewing is illegal in KSA, the practice is widespread and deeply rooted; this may be attributed to Jazan's location adjacent to Yemen, where khat is cultivated and is used by two thirds of Yemenis [3].

The main objective of this study is to evaluate the association between adolescents' khat chewing behavior and characteristics of families in Jazan in the Kingdom of Saudi Arabia. We investigate the family characteristics of parental khat chewing behavior as well as siblings' khat abuse. This study also tests the association between adolescents' khat chewing behavior and parental socioeconomic status, as measured by education levels and family income.

## Material and methods

### Study design

Our observational cross-sectional survey targeted school students at the intermediate and secondary school levels in Jazan. The inclusion criteria for the study sample were full-time student status, enrollment in one of Jazan's intermediate or secondary schools during the academic year 2011–2012, and age of 13–21 years. General education in KSA is divided into a pre-education stage, where children aged 3–5 years go to kindergarten, and primary education, in which children enter at the age of 6 and remain for 6 years. Intermediate education in Saudi Arabia lasts 3 years, followed by a final 3 years of secondary education.

### Study setting

Jazan (also called Gizan) is one of the 13 provinces of the Kingdom of Saudi Arabia. It is located on the tropical Red Sea coast in southwestern Saudi Arabia and covers an area of 11,671 km^2^, including some 5,000 villages and towns. Attached to it are 100 islands, including the largest, Farasan. Jazan runs along the Red Sea's coast for almost 300 km and is highly populated, with 1.5 million residents [18].

### Sample size and design

A representative sample was determined to be 4,100 students, calculated on a large khat survey conducted in 2005 ((prevalence 21.4%), 95% confidence interval (CI), marginal error 2% and non-response rate 10%, design effect 1.5) [17]. The sampling design was a random cluster sampling in three stages based on educational sectors, schools, and classes. Two intermediate schools and two secondary schools for each gender were selected from the nine educational sectors of Jazan. A total of 72 intermediate and secondary schools in Jazan were selected using systematic random sampling. The sampling frames for school selection and the study participants were prepared in consultation with the Ministry of Education, the regional education directorate, and respective schools (to obtain details of classes and number of students in each class level). Probability proportional to size sampling was used to determine the number of students in the selected schools. Systematic random sampling was used to select target students from classes within each school.

### Data collection

A standardized, self-administered questionnaire was used for data collection and was modified to suit schools' student populations. A pilot study was carried out on 160 students to fine-tune questions and to assess the study instrument's reliability before initiating actual data collection. After the pilot study was conducted, minor modifications were made to the original questionnaire. The final questionnaire contained 80 multiple-choice questions organized in four sections. Questions covered demographic data (age, sex) and family characteristics, parents' khat chewing status, parents' attitudes toward their children's khat chewing, khat chewing among siblings, and khat chewing and smoking among peers. The questionnaire asked whether pupils had tried chewing khat at least once in their lifetime, whether they had tried it in the last month, and from where the student had obtained khat during the past month (and if so, how many days they had chewed). The first question was used to evaluate the lifetime prevalence of khat use, whereas the other questions were used to calculate the current prevalence of khat chewing. Permission was obtained from school headmasters and class instructors to collect data during classes. The questionnaires were distributed and collected by health workers from the school health directorate. The anonymity of participants was emphasized, and confidentiality was strictly maintained on all collected questionnaires.

We used the following operational definitions: (a) non-khat user, students who have never used khat in any form; (b) current prevalence of chewing, the proportion of the study population who were chewing khat within 30 days preceding the study; and (c) ever chewer, an individual who has chewed even if only once in his/her lifetime.

### Statistical analysis

Data entry took place in the Substance Abuse Research Center, Jazan University under the supervision of a data analysis specialist. The data entry and analysis were performed using Epi-info (version 3.5.3) and SPSS (version 17) software. To ensure data entry quality, double data entry was performed in eight randomly selected schools and produced good results. Data analysis involved descriptive statistics as well as inferential statistics. Simple tabulation frequencies were used to give a general overview of the data. Khat chewing prevalence was presented using 95% CIs; chi-squared or Fisher exact tests were performed to determine the associations between individual categorical variables and the outcome (khat chewing). The multivariate analysis involved the spontaneous selection of variables significantly associated with khat chewing by a backward stepwise technique fitted in a multivariate logistic regression model. The final model of factors was checked for fitness using the Hosmer-Lemeshow goodness of fit test. A final regression model was also analyzed for all possible two-way interactions and revealed no significant interaction in the final model. All statistical analyses were performed at the 95% confidence level.

### Ethical issues

The study proposal and instruments were approved by Jazan University's review board, and voluntary informed oral consent was obtained from each student enrolled in the study.

## Results

The response rate for distributed questionnaires was 95.68% (3,923 from the target of 4,100 students). The response rates for answering particular questionnaire questions varied, as some students left out some items. The mean age of the participants was 15 years old (SD = 2.01). As seen in Table 1, most sampled students (75.1%) belonged to the age group of 15–19 years. The distribution of students shows that 61.3% of students were from urban areas and 38.7% came from rural areas. As planned during sampling, approximately 57.3% of the students were from upper secondary schools and 42.7% from intermediate schools. The gender distributions show that 56.3% of the students were males and 43.7% were females.

**Table 1:** Background characteristics of the study population

**Characteristics**	**Intermediate**		**Secondary**		**Total**
	**Male**	**Female**	**Male**	**Female**	
Age groups (years)					
10–14	415 (44.6)	450 (56.1)	10 (0.8)	27 (2.8)	872 (22.2)
15–19	515 (55.4)	328 (43.9)	1,199 (93.3)	909 (93.8)	2,951 (75.1)
20–21	-	-	76 (5.9)	33 (4.4)	109 (2.8)
Mode of living					
Rural	298 (32.0)	320 (42.8)	543 (42.3)	360 (37.2)	1,521 (38.7)
Urban	632 (68.0)	428 (57.2)	742 (57.7)	609 (62.8)	2,411 (61.3)
Total	930 (100)	748 (100)	1,285 (100)	969 (100)	3,923 (100)
	1,678 (42.7)		2,254 (57.3)		

**Figure 1:**
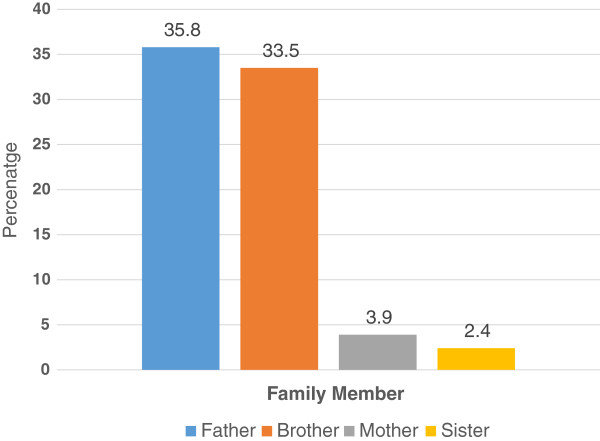
Lifetime khat chewing among family members.

As shown in Table 2, the prevalence of current khat chewing among students was 20.5% (95% CI 19.27–21.79) and was significantly higher for males 33.1% (95% CI 31.16–35.08) than for females 4.3% (95% CI 3.39–5.31) (*P* < 0.001). Ever khat chewer students accounted for 24.2% (95% CI 22.9–25.57) of the population sampled, 38.8% (95% CI 36.82–40.88) of whom were males, which is a significantly higher percentage than the 5.4% (95% CI 4.39–6.53) of ever khat chewers who were females (*P* < 0.001). The current khat chewing prevalence among intermediate schools was 16.2%, which is significantly lower than that at upper secondary schools, where it was 23.7% (*P* < 0.001). The same indicators were 22.6% for rural students and 19.2% for urban students. Ever khat chewing prevalence was found to be 19.9% in intermediate schools and 27.4% in secondary schools. Turning to family influences, Figure 1 shows khat chewing habits in students' families; khat chewing lifetime prevalence among fathers, brothers, mothers, and sisters was found to be 35.8% (95% CI 34.3–37.2), 33.5% (95% CI 32.0–35.0), 3.9% (95% CI 3.3–4.6), and 2.4% (95% CI 2.0–2.9), respectively.

**Table 2:** Prevalence of khat chewing among students

**Category**	**Current khat chewers**		**Ever khat chewers**	
	**No. (%)**	**95% CI**	**No. (%)**	**95% CI**
Gender				
Male	733 (33.1)***	31.16–35.08	860 (38.8)***	36.82–40.88
Female	73 (4.3)	3.39–5.31	92 (5.4)	4.39–6.53
School level				
Intermediate schools	272 (16.2)**	14.52–18.05	334 (19.9)**	18.06–21.88
Secondary schools	534 (23.7)	21.98–25.49	618 (27.4)	25.62–29.30
Mode of living				
Urban	462 (19.2)*	17.62–20.78	551 (22.9)*	21.22–24.57
Rural	344 (22.6)	20.59–24.79	401 (26.4)	24.21–28.63
Total	806 (20.5)	19.27–21.79	952 (24.2)	22.9–25.57

*P* value indicates an independent statistical testing. *P < 0.05; **P < 0.01; ***P < 0.001.

Table 3 provides the percentage of students who chew khat associated with selected family characteristics. From the table, there was no significant variation in khat chewing prevalence among males and females according to parental (both paternal and maternal) education. There was also no significant difference between a student's khat use and the existence of family disputes for both males and females. Family income levels were not associated with khat chewing prevalence among the adolescents.

**Table 3:** Prevalence of khat chewing according to certain family characteristics

**Characteristics**	**Gender**^**a**^		**Total**
	**Male (*n*** **= 860)**	**Female (*n*** **= 92)**	
Father's education			
Illiterate	142 (35.8)	11 (5.1)	153 (25.0)
Primary	141 (42.8)	13 (5.3)	159 (27.0)
Intermediate	126 (42.9)	9 (4.2)	135 (26.6)
Secondary	102 (39.1)	12 (4.7)	114 (22.1)
University and above	171 (34.1)	25 (5.3)	196 (20.2)
Mother's education			
Illiterate	252 (37.2)	24 (4.8)	281 (23.5)
Primary	163 (40.8)	23 (6.7)	186 (25.1)
Intermediate	102 (44.7)	13 (7.3)	115 (28.3)
Secondary	63 (36.6)	95 (3.3)	68 (20.9)
University and above	115 (33.9)	12 (3.8)	127 (19.5)
Family disputes			
None	557 (39.2)	30 (4.4)	587 (27.8)
Rarely	143 (36.0)	26 (5.3)	169 (19.1)
Sometimes	93 (41.7)	17 (4.2)	110 (17.5)
Always	21 (50.0)	16 (19.5)	37 (29.8)
Family income			
Less than $1,000	160 (41.5)	10 (3.2)	170 (24.4)
$1,001–3,000	247 (40.2)	22 (4.4)	269 (24.2)
More than $3,000	158 (35.9)	18 (6.3)	176 (24.2)

^a^No significant association was observed.

Table 4 shows family attitudes toward khat use habits for both sexes. For example, 31.9% of students reported that their fathers agreed with their khat use behavior (there was no significant difference between males and females). Moreover, 24.0% of them reported that their mothers approved of their khat chewing behavior. Surprisingly, 38% of fathers and 36.9% of mothers did not know about their children's khat use. When students reported with whom they chewed khat, 60.2% of males said they chewed with friends, while 32.9% said they chewed with their families (there were significant differences between their responses (*P* < 0.001)). The table also shows that 39.5% of adolescents chewed khat for the first time with their friends, while 26.7% tried khat for the first time alone, with a significant difference occurring between the sexes (*P* < 0.001).

**Table 4:** Family attitude toward khat chewing

**Characteristics**	**Male**	**Female**	**Total**	***P *value**
	**(*n*** **= 860)**	**(*n*** **= 92)**		
Father's attitude toward khat chewing				*P* > 0.05
Agree	255 (32.2)	22 (28.2)	277 (31.9)	
Disagree	183 (23.1)	20 (25.6)	203 (23.4)	
Do not know	299 (37.8)	31 (39.7)	330 (38.0)	
Father dead	54 (6.8)	5 (6.4)	59 (6.8)	
Mother's attitude toward khat chewing				*P* > 0.05
Agree	185 (23.5)	22 (28.2)	207 (24.0)	
Disagree	291 (37.0)	26 (33.3)	317 (36.7)	
Do not know	292 (37.2)	27 (34.6)	319 (36.9)	
Mother dead	18 (2.3)	3 (3.8)	21 (2.4)	
With whom do you chew khat				
Family	45 (5.8)	23 (32.9)	68 (8.1)	*P* < 0.001
Relatives	152 (19.7)	13 (18.6)	165 (19.6)	
Friends	464 (60.2)	16 (22.9)	480 (57.1)	
Alone	110 (14.3)	18 (25.7)	128 (15.2)	
With whom did you chew khat for the first time				*P* < 0.001
Father/mother	90 (11.5)	11 (15.9)	101 (11.9)	
Relative	163 (20.9)	23 (33.3)	186 (21.9)	
Friends	322 (41.2)	14 (20.3)	336 (39.5)	
Alone	206 (26.4)	21 (30.4)	227 (26.7)	

Family variables such as family members' khat use status were found to be highly associated with students' khat use (*P* < 0.001 for all). Having many close friends who chew khat or smoke and smoking status of the student himself or herself were also positively associated with a student's khat chewing status. The student's residential patterns are also an important factor that can be associated with khat use (*P* < 0.05). Those who lived with their parents had the lowest khat chewing percentage at 23.4% compared with the 43.3% of khat chewers who lived alone (Table 5).

**Table 5:** Comparison of khat chewers versus non-chewers with regard to certain variables

**Variables**	**Khat chewing status**		***P *value**
	**Khat chewers**	**Non-chewers**	
Father using khat (*n* = 3,557)			*P* < 0.001
Yes	461 (32.8)	945 (67.2)	
No	397 (18.5)	1,754 (81.5)	
Mother using khat (*n* = 3,699)			*P* < 0.001
Yes	62 (40.3)	92 (59.7)	
No	821 (23.2)	2,724 (76.8)	
Brother using khat (*n* = 3,635)			*P* < 0.001
Yes	487 (37.9)	830 (63.0)	
No	372 (16.0)	2,776 (84.0)	
Sister using khat (*n* = 3,682)			*P* < 0.001
Yes	48 (50.5)	47 (49.5)	
No	829 (23.1)	2,758 (76.9)	
Student smoking status (*n* = 3,628)			*P* < 0.001
Yes	489 (54.3)	138 (5.1)	
No	411 (45.7)	2,590 (94.9)	
Friends smoking (*n* = 3,667)			*P* < 0.001
Yes	592 (66.0)	689 (24.9)	
No	305 (34.0)	2,083 (75.1)	
Friend using khat (*n* = 3,761)			*P* < 0.001
Yes	746 (81.2)	762 (26.8)	
No	173 (18.8)	2,080 (73.2)	
Residence pattern (*n* = 3,894)			*P* < 0.05
Parents	815 (23.4)	2,670 (76.6)	
Father	31 (36.5)	54 (63.5)	
Mother	63 (26.8)	172 (73.2)	
Relatives	19 (32.2)	40 (67.8)	
Alone	13 (43.3)	17 (56.7)	

The results of the univariate and multivariate logistic regression analyses for potential risk factors of khat chewing are shown in Table 6. Univariate analysis revealed that family members' khat use status, having close friends who chew khat or smoke, and student smoking status were associated with a significant risk of khat chewing (*P* < 0.001 for all). The multivariate logistic regression analysis suggests that the most important independent predictors of khat chewing among the students in our sample were students' smoking status (odds ratio (OR) = 14.03, *P* < 0.001), friends using khat (OR = 5.652, *P* < 0.001), sister's use of khat (OR = 2.04, *P* < 0.05), father's use of khat (OR = 1.45, *P* < 0.001), and brother's use of khat (OR = 1.56, *P* < 0.05).

**Table 6:** Univariate and multivariate logistic regression analyses for family khat use-related factors among study participants

**Category**	**Univariate**			**Multivariate**^**a**^		
	**OR**	**95% CI**	***P *value**	**OR**	**95% CI**	***P *value**
Mother using khat						
No (ref.)	1					
Yes	2.24	1.61–3.11	*P* < 0.001			
Father using khat						
No (ref.)	1			1		
Yes	2.15	1.84–2.51	*P* < 0.001	1.45	1.16–1.82	*P* < 0.001
Brother using khat						
No (ref.)	1			1		
Yes	3.07	2.62–3.59	*P* < 0.001	1.56	1.27–2.00	*P* < 0.05
Sister using khat						
No (ref.)	1			1		
Yes	3.30	2.52–5.11	*P* < 0.001	2.041	1.11–3.74	*P* < 0.05
Student smoking status						
No (ref.)	1			1		
Yes	22.33	17.99–27.70	*P* < 0.001	14.03	10.76–18.30	*P* < 0.001
Friends smoking						
No (ref.)	1			1		
Yes	5.87	4.99–6.91	*P* < 0.001	1.44	1.08–1.94	*P* < 0.05
Friend using khat						
No (ref.)	1			1		
Yes	11.77	9.78–14.16	*P* < 0.001	5.65	3.92–8.14	*P* < 0.001

^a^Hosmer-Lemeshow goodness of fit test *χ*^2^ = 7.01, *p* = 0.43.

## Discussion

The objective of this study was to evaluate the role of family factors like parental education levels, family members' khat use, and other factors on the khat chewing status of adolescents in the province of Jazan in KSA.

The study revealed that a significant proportion of Jazan students (20.5%) chew khat (33.1% for males and 4.3% for females). The prevalence of khat chewing among students is less than that observed among the general population in Jazan, Yemen, Ethiopia, and Somalia [19,20]. Lifetime khat chewing prevalence in the current study was 24.2% (males 38.8%, females 5.4%), which is approximately similar to that reported in Jazan previously [16,17]. The use of khat (current and lifetime) was significantly higher among males, which also parallels results reported previously in Jazan and Ethiopia [16,17].

Unlike the bulk of the tobacco literature where the association between socioeconomic status and smoking is well established [21-23], parental education levels, occupation, and family income levels have no significant association with adolescents' khat chewing behavior. This is similar to findings on the roles of family backgrounds on cigarette smoking among adolescent school children in Slovakia, where parent educational level and employment status were not statistically associated with students' smoking status [24].

Social acceptability of khat chewing and socialization of this habit increase the likelihood of adolescents adopting the behavior. Less than a quarter of the study population reported that their parents disagree with their use of khat. This attitude is further supported by the socialization of this habit, as more than half of the female students and a quarter of the male students chew khat with family members and relatives. This social acceptability is more pronounced when we consider circumstances in which the adolescent chewed khat for the first time. Again, about half of the female students and a third of the male students chewed khat for the first time with family members and relatives. In general, the majority of adolescents reported that they use khat with someone else rather than chewing alone. This finding is consistent with the broader literature on adolescent substance use in general [25] and adolescent smoking in particular [26]—adolescents who receive low levels of parental behavioral control and acceptance show the greatest prevalence of substance abuse [9]. It is equally important that 38.0% of students said that their fathers were unaware of their khat use, compared with 36.9% who reported that their mothers were unaware. These figures testify to the significant number of adolescents in Jazan who chew khat without their parents' knowledge.

Residence patterns also appear to impact khat chewing status. This study suggested that adolescents living with their parents have the lowest percentage of khat chewing at 23.4% compared with the 43.3% of khat chewers who live alone. Similar findings with alcohol use have been reported in the USA [8].

Knowledge on risk factors and predictors is crucial to designing prevention programs that target high-risk groups of adolescents. Family members' khat chewing status, having close friends who chew khat or smoke, and student smoking status were highly associated with a significant risk of khat chewing. The associations between friends' and adolescents' substance abuse behaviors have been deeply investigated in the literature [27-31]. Our univariate analysis documented the strong impact of peers on khat chewing status. Regarding the impact of family khat use, the most important independent predictors of khat chewing among students in our sample were a sister using khat, a father using khat, and a brother using khat. The substance abuse literature suggests that parental abuse is an influential factor affecting children's substance abuse status. Other studies have found both parental smoking behavior and parents' attitudes toward their children's smoking are influential factors for adolescent smoking [32-34].

The main strength of this study lies in the fact that it is the first study to investigate the role of family characteristics in khat chewing in the Jazan region. However, some significant limitations should be mentioned. The obvious limitations include the fact that the questionnaire given to students at the intermediate level was self-administered. Data collected based on student self-reports may be subject to recall bias and to under-reporting of khat use due to a social desirability bias. Additionally, the cross-sectional study design may not be suitable for assessing directional and causal relationships of the studied variables with khat chewing behavior. Furthermore, the reporting of family khat use was based on lifetime khat chewing only. Finally, the study assessed only associations between family variables and khat use, with no emphasis given to khat use patterns and excessive use. In summary, our study calls for more in-depth research on the relationship between family khat chewing and adolescent khat chewing behavior.

## Conclusion

The study results highlight the significant impact of peers, fathers, sisters, and brothers on adolescent risk-taking behaviors and the need to strengthen the family's role in prevention. Khat is a socially acceptable habit in Jazan, and our findings suggest that khat control program efforts need to focus on peers and family members to reduce the prevalence of the habit and its unfavorable consequences. Peer impact is higher among male users than among females. With both genders, these results emphasize the importance of peer education interventions as one strategy for changing social norms in the population.

## Competing interests

The authors declare that they have no competing interests.

## Authors’ contributions

MSM, RMA, and AMG prepared the project proposal and designed the research paper. MSM and AMG performed data analysis. MSM, AMG, and RMA wrote the manuscript and provided significant input on the manuscript. All authors read and approved the final manuscript.
